# Expression levels of TWIST1 are associated with the clinicopathological stage of B-cell non-Hodgkin lymphoma

**DOI:** 10.3892/etm.2014.1962

**Published:** 2014-09-15

**Authors:** CUNDONG JIA, LIPING LIANG, LILI YANG, FENG ZHAO, JINGPING BAI

**Affiliations:** 1 Department of Medical Oncology, The Affiliated Tumor Hospital of Xinjiang Medical University, Ürümqi, Xinjiang 830011, P.R. China; 2 Department of Pathology, The Affiliated Tumor Hospital of Xinjiang Medical University, Ürümqi, Xinjiang 830011, P.R. China; 3 Department of Bone Oncology, The Affiliated Tumor Hospital of Xinjiang Medical University, Ürümqi, Xinjiang 830011, P.R. China

**Keywords:** B cell non-Hodgkin, lymphoma, TWIST1, immunohistochemistry staining

## Abstract

The aim of the present study was to investigate the expression level of TWIST1 in B-cell non-Hodgkin lymphoma (BNHL) and its association with the clinicopathological characteristics of BNHL. Expression levels of TWIST1 were analyzed in patients with BNHL (n=45) and lymphadenosis (n=21) using immunohistochemical staining and western blot analysis. In addition, the mRNA expression levels of TWIST1 in the peripheral blood were detected by fluorescent quantitative polymerase chain reaction. The positive rate of TWIST1 expression in the BNHL tissue was 82.2%, which was significantly higher compared with the lymphadenosis tissue (5%; P<0.05). In addition, the protein expression level of TWIST1 in the BNHL tissue was higher compared with the lymphadenosis tissue. TWIST1 expression was also higher in stage III/IV BNHL tissues than in stage I/II tissues (P<0.05). The tissues were staged following the Ann Arbor system. Furthermore, the mRNA expression level of TWIST1 in the peripheral blood of the BNHL tissue (3.03±0.03) was higher compared with the lymphadenosis tissue, and the mRNA expression level of TWIST1 was higher in stage III/IV (4.41±0.12) tissues than in stage I/II BNHL (2.03±0.08) tissues. In conclusion, TWIST1 expression was higher in the tissue and peripheral blood of patients with BNHL when compared with those with lymphadenosis. Thus, TWIST1 expression was associated with the clinicopathological stage of BNHL.

## Introduction

B-cell non-Hodgkin lymphoma (BNHL) refers to a group of malignant tumors caused by malignant B cell monoclonal expansion (1). The main clinical treatment for BNHL is chemotherapy (2–4); however, the prognosis rate is poor. Currently, bone marrow biopsy is the most reliable method for the clinical diagnosis of BNHL (5,6). However, the specific indicators for BNHL diagnosis and prognosis remain unclear (7,8).

The TWIST1 gene, a highly conserved transcription factor, belongs to an alkaline helix-loop-helix protein family and plays an important role in embryo development. Furthermore, TWIST1 is associated with tumorigenesis, cell proliferation and cell differentiation (9). Previous studies have revealed that the TWIST1 gene regulates cell apoptosis and promotes epithelial-mesenchymal transition via the extracellular signal-regulated kinase-1/2 (10) and Akt signaling pathways (11,12). BNHL may be induced through an imbalance between the proliferation and apoptosis of B cells (13). TWIST1 has been demonstrated to not only affect tumor cell apoptosis, but also promote the invasion and migration of various tumors (14). However, the role of TWIST1 expression in BNHL has not yet, to the best of our knowledge, been studied.

In the present study, the expression levels of TWIST1 in the tissues and peripheral blood of 45 cases of BNHL and 21 cases of lymphadenosis were detected using immunohistochemistry, western blot analysis and fluorescent quantitative polymerase chain reaction (PCR). In addition, the association between TWIST1 expression in the peripheral blood of BNHL patients and the clinicopathological characteristics of BNHL were further analyzed.

## Materials and methods

### Clinical data of the patients

In total, 45 patients that had been diagnosed with BNHL at The Affiliated Tumor Hospital of Xinjiang Medical University (Ürümqi, China) between December 2011 and December 2012 were enrolled in the study. Tissue samples and peripheral blood were collected from each patient. Among the 45 patients with BNHL, there were 26 males and 19 females. The age of the patients ranged between 15 and 68 years, with a median age of 45 years and a mean age of 42.5 years. According to the World Health Organization classification of lymphoid neoplasms (15), there were 37 cases of diffuse large B-cell lymphomas and eight cases of mantle cell lymphoma. According to the Ann Arbor staging system (16), 26 cases of BNHL were in stage III/IV and 19 cases were in stage I/II. For the control group, tissue samples and peripheral blood were collected from 21 patients with lymphadenosis. Of these, 14 were male and seven were female, with a mean age of 35 years and a median age of 37 years.

Prior written and informed consent was obtained from each patient and the study procedure was approved by the Ethics Review Board of The Affiliated Tumor Hospital of Xinjiang Medical University.

### Reagents

A rabbit anti-human TWIST1 polyclonal antibody was purchased from Abcam (Burlingame, CA, USA) and an S-P immunocytochemical assay kit was purchased from Beijing Zhongshan Golden Bridge Biotechnology Co., Ltd. (Beijing, China). An EasySpin rapid whole blood RNA extraction kit (spin-column) was obtained from Biomed Biotech Company (Beijing, China) and a Reverse Transcription system was purchased from Chengdu Bo Ruike Biological Company (Chengdu, China). SYBR Green Real-Time PCR reagents were obtained from Kapa Biosystems (Boston, MA, USA).

### Immunohistochemistry

All the tissue samples were surgically removed and immediately frozen in liquid nitrogen. Immunohistochemistry was performed with a kit, according to the manufacturer’s instructions. Briefly, tumor tissues were fixed with 10% formalin and embedded in paraffin. The tissue samples were cut into a series of 4-μm conventional sections, which were subsequently dewaxed in serial dilutions of dimethylbenzene and dehydrated in ethanol. Endogenous peroxidase was inactivated with freshly prepared hydrogen peroxide (3%) at room temperature for 10 min. Phosphate-buffered saline (PBS) was heated using a microwave to repair the antigen. The sections were incubated with a rabbit anti-human polyclonal anti-TWIST1 antibody for 1 h at room temperature. Subsequently, a biotinylated secondary antibody immunoglobulin G was added and incubated at 37°C for 30 min, and 3,3′-diaminobenzidine chromogenic reagent was used for color development. The sections were subsequently counterstained with hematoxylin. Following hydrochloric acid differentiation and dimethylbenzene transparency, sections were mounted with neutral gum.

### Blood sample preparation

Venous blood (5 ml) was collected from the patients with BNHL and lymphadenosis using a vacuum blood collection tube. The blood was centrifuged at 1,600 × g for 10 min, and the precipitate was collected for RNA extraction.

### Total RNA extraction

Total RNA was extracted using the EasySpin rapid whole blood RNA extraction kit, in strict accordance with the manufacturer’s instructions. The absorbance ratio of RNA at 260/280 nm was determined using RNA electrophoresis and a spectrophotometer (Bio-Rad Laboratories, Hercules, CA, USA) in order to determine the weight of the RNA. A total of 1 μg RNA was used for the reverse transcription reaction. An oligo-dT 18 primer, which was part of the reverse transcription system was used (FSK 201). The reverse transcribed cDNA was stored at −20°C.

### Western blot analysis

For sample preparation, 100 mg BNHL tissue was pulverized in liquid nitrogen to obtain a fine power and lysed in radioimmunoprecipitation assay lysis buffer (with protease inhibitor) at 4°C overnight. Following centrifugation at 14,000 × g for 10 min at 4°C, the supernatant was collected. After adding 2× sample buffer, the proteins in the supernatant were denatured by boiling at 95°C for 5 min. The protein samples (10 μl) were separated using SDS-PAGE and transferred onto a polyvinylidene fluoride membrane. Following blocking with skimmed milk at room temperature for 1 h, an anti-TWIST1 primary antibody (1:500) was added and incubated at 4°C overnight. Following rinsing three times with PBS Tween-20 (PBST), a horseradish peroxidase-conjugated secondary antibody (Abcam). was added and incubated at room temperature for 1 h. The membranes were rinsed three times again with PBST, and subsequently developed with an enhanced chemiluminescence reagent. β-actin (Abcam )was used as an internal control.

### Fluorescent quantitative PCR

Quantitative PCR was performed using SYBR Green Real-Time PCR reagents and a ABI 7300 thermal cycler (Applied Biosystems, Foster City, CA, USA) was used. The reaction system consisted of 10 μl qPCR mix, 0.5 μl upstream primer, 0.5 μl downstream primer, 1 μl cDNA and 8 μl ddH_2_O. Three parallel wells were used for each sample. The procedures for the PCR reaction were predenaturation at 95°C for 10 min, 40 cycles of denaturation at 95°C for 1 min, annealing at 58°C for 30 sec, extension at 72°C for 30 sec and a final extension at 72°C for 3 min.

### Evaluation of the immunohistochemical results

Slices were observed at ×400 magnification and positive cells were defined as cells with brown staining in the tumor cell cytoplasm or membrane. Five representative high power fields were randomly selected. The ratio of the positive cell number to the total number of tumor cells was considered as the percentage of stained cells, and the average percentage of stained cells from the five fields was defined as the positive cell percentage. Based on the percentage of positive staining, immunohistochemical staining results were scored as follows: 0, 0% positive staining; 1, 1–25% positive staining; 2, 26–50% positive staining and; 3, 51–100% positive staining. Based on the staining intensity, immunohistochemical staining results were scored as follows: 0, no staining; 1, light yellow; 2, brownish-yellow and; 3, brown. The degree of staining was calculated by combining the percentage of positive staining and the intensity of staining. The overall degree of staining was defined as follows: 0–1, negative staining; 2–3, weak positive staining; 4–6, medium positive staining and; >6, strongly positive staining.

### Statistical analysis

SPSS 16.0 statistical software (SPSS, Inc., Chicago, IL, USA) was used for statistical analysis. Data are presented as the mean ± standard deviation. TWIST1 expression in the peripheral blood was compared between groups using the t-test, while the correlations between TWIST1 protein expression and pathological features were analyzed using the χ^2^ test. P<0.05 was considered to indicate a statistically significant difference.

## Results

### TWIST1 protein expression is higher in BNHL tissue when compared with lymphadenosis tissue and is associated with the clinical stage of BNHL

**Figure 1:**
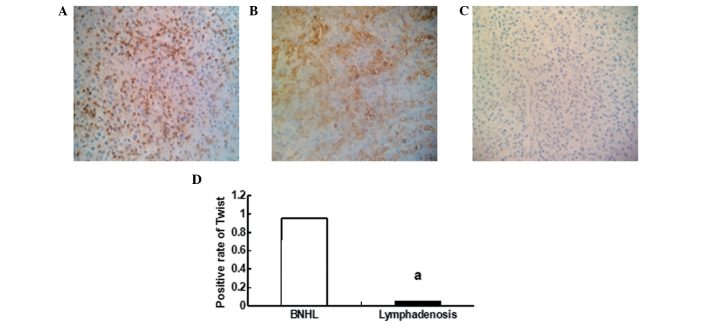
Expression levels of TWIST1 in BNHL and lymphadenosis tissues (magnification, ×10). (A) Strong positive expression in the cytoplasm. (B) Strong positive expression in the cell membrane. (C) Negative expression in the lymphadenosis group. (D) Positive rate of TWIST1 expression in the BNHL tissue. BNHL, B-cell non-Hodgkin lymphoma. Immunohistochemical staining was performed to detect the expression levels of TWIST1. ^a^P<0.05 vs. the BNHL tissue.

To determine the protein expression levels of TWIST1, immunohistochemical staining was performed in 45 patients with BNHL and 21 patients with lymphadenosis. Representative immunohistochemical staining results are shown in Fig. 1. TWIST1 was predominantly expressed in the cytoplasm and on the cell membrane. Fig. 1A shows the strong positive expression of TWIST1 in the cytoplasm of cells in the BNHL tissue. In addition, Fig. 1B shows the strong positive expression of TWIST1 in the cell membranes of cells in the BNHL tissue. Among the 45 cases of BNHL, 8 cases (17.8%) exhibited negative TWIST1 expression, 15 cases (33.3%) exhibited weak positive TWIST1 expression and 22 cases (48.9%) exhibited mild and strong positive TWIST1 expression. In the 15 cases of weak positive TWIST1 expression, 13 cases were at BNHL stage I/II. In the 22 cases of mild and strong positive TWIST1 expression, 20 cases were at BNHL stage III/IV. Fig. 1C shows the negative expression of TWIST1 in the cytoplasm of cells in the lymphadenosis tissue. Among the 21 patients with lymphadenosis, there was one case (5%) that exhibited weak positive expression of TWIST1 and 20 cases of negative TWIST1 expression. Correlation analysis between TWIST1 expression and the clinicopathological features of patients with BNHL was performed. As shown in Table I, TWIST1 expression was significantly associated with the clinical stage (P<0.05); however, no associations were observed with regard to gender, age or pathological subtype. Fig. 1D shows the positive rate of TWIST1 expression in the BNHL and lymphadenosis tissues.

**Figure 2:**
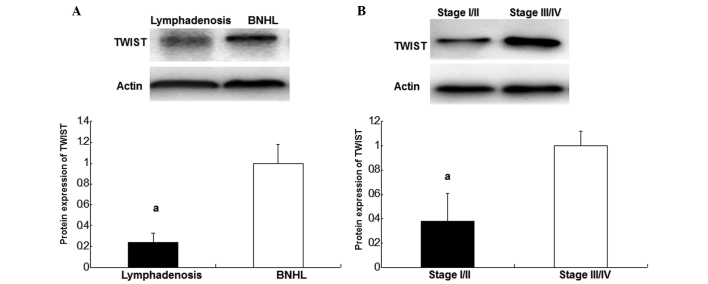
TWIST1 protein expression was analyzed using western blot analysis. (A) Protein expression of TWIST1 in BNHL and lymphadenosis tissues ^a^P<0.05 vs. the BNHL tissue. (B) Protein expression of TWIST1 in BNHL tissue at various stages. BNHL, B-cell non-Hodgkin lymphoma. ^a^P<0.05 vs. the stage III/IV BNHL tissue.

Western blot analysis was conducted to further verify the expression levels of TWIST1. As shown in Fig. 2, TWIST1 expression in the BNHL tissue was significantly higher compared with the lymphadenosis tissue (P<0.05). Furthermore, TWIST1 expression in stage III/IV BNHL tissues was higher compared with stage I/II BNHL tissue; this difference was statistically significant (P<0.05). These observations indicated that TWIST1 expression may be associated with tumor progression in BNHL (Fig. 2). Thus, TWIST1 may play an important role in the tumor progression of BNHL.

### TWIST1 mRNA expression is higher in the peripheral blood of patients with BNHL

**Figure 3:**
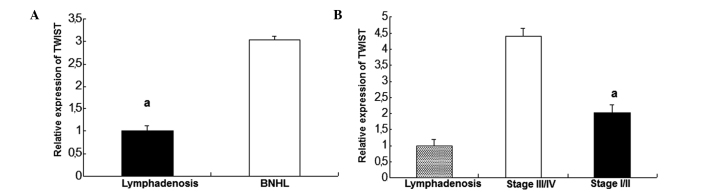
Expression levels of TWIST1 in the peripheral blood were detected using SYBR Green fluorogenic quantitative polymerase chain reaction. (A) Relative expression levels of TWIST1 in the BNHL and lymphadenosis tissues ^a^P<0.05 vs. the BNHL tissue. (B) Relative expression levels of TWIST1 in the lymphadenosis and BNHL tissues at various stages. BNHL, B-cell non-Hodgkin lymphoma. ^a^P<0.05 vs. the stage III/IV BNHL tissue.

To detect the mRNA expression levels of TWIST1, quantitative PCR was conducted on the peripheral blood of the 45 patients with BNHL and 21 patients with lymphadenosis. Quantitative results are shown in Fig. 3. As shown in Fig. 3A, the mRNA expression levels of TWIST1 in the peripheral blood of BNHL patients was significantly higher (3.03±0.03) compared with the patients with lymphadenosis; this difference was statistically significant (P<0.05). In addition, the mRNA expression levels of TWIST1 at the various clinical stages of BNHL were further analyzed. As shown in Fig. 3B, the mRNA expression of TWIST1 at stage III/IV BNHL (4.41±0.12) was higher compared with stage I/II BNHL (2.03±0.08); this difference was also statistically significant (P<0.05). These results indicated that the mRNA expression level of TWIST1 is higher in BNHL tissue than in lymphadenosis tissue, and increases with increasing BNHL clinical pathological stage.

## Discussion

BNHL is a common hematopoietic malignancy that predominantly originates from the lymph nodes and lymph tissue. The malignancy progresses rapidly and is highly invasive (17). BNHL is a heterogeneous tumor and is mainly caused by the disturbance of signaling pathways in B cells (10,12). TWIST1 is a critical gene in embryo development (18); however, the role of TWIST1 in the development of BNHL has not yet, to the best of our knowledge, been studied. In the current study, tissue and peripheral blood samples from clinical patients with BNHL were collected. TWIST1 expression was determined by immunohistochemistry, western blot analysis and quantitative PCR. In addition, the association between TWIST1 expression and BNHL tumor progression was further analyzed.

The results revealed that TWIST1 was highly expressed in the BNHL tumor tissue. Immunohistochemical analysis demonstrated that the TWIST1 protein was primarily distributed in the cytoplasm and cell membrane. The positive rate of TWIST1 expression in the BNHL tissues was 92% (41/45) and only 5% (1/21) in the lymphadenosis tissue; the difference between the two groups was statistically significant (P<0.05). BNHL cases of stage III/IV had a higher positivity rate of TWIST1 expression compared with BNHL of stage I/II, indicating that TWIST1 expression increased gradually with the progression of BNHL. Similarly, western blot analysis revealed that the protein expression level of TWIST1 was higher in the BNHL tissues than in the lymphadenosis tissues; the difference between the two groups was statistically significant (P<0.05). TWIST1 expression in the BNHL stage III/IV cases was higher compared with the stage I/II tissues. This result was consistent with the immunohistochemical results, which further verified that TWIST1 was involved in the development of BNHL. The χ^2^ test demonstrated that there were no statistically significant differences in TWIST1 expression with regard to gender, age and pathological subtypes. The quantitative PCR results revealed that the mRNA expression levels of TWIST1 in the peripheral blood of patients with BNHL were significantly higher (P<0.05) compared with those in the peripheral blood of patients with lymphadenosis. In addition, the mRNA expression level of TWIST1 in patients with BNHL at stage III/IV was significantly higher compared with those at stage I/II (P<0.05). The results demonstrated that TWIST1 mRNA expression increased gradually with the development of BNHL. Thus, TWIST1 may play an important role in the malignant transformation of B cells and be used as a biomarker for the clinical diagnosis and prognosis of BNHL. However, the underlying mechanisms of TWIST1 in BNHL progression require further investigation.

In conclusion, TWIST1 expression was shown to be associated with the progression of BNHL. Thus, TWIST1 may be used for predicting the recurrence and metastasis of BNHL, and as a novel target for the diagnosis and treatment of BNHL.

## Figures and Tables

**Table I:** Correlation between TWIST1 expression and the clinicopathological features of patients with BNHL.

Variables	Cases of (n)	Positive rate TWIST1 expression	χ^2^-value	P-value
Gender				
Male	26	20/26	0.005	1.000
Female	19	17/19		
Age (years)				
>60	14	13/14	0.012	1.000
≤60	31	24/31		
Pathological subtype				
DLBCL	37	32/37	0.028	1.000
ML	8	5/8		
Stage				
III/IV	26	22/26	7.440	0.026
I/II	19	15/19		

BNHL, B-cell non-Hodgkin lymphoma; DLBCL, diffuse large B-cell lymphoma; ML, mantle cell lymphoma.
